# Gingival Enlargement in a Case of Variant Jones Syndrome: a Case Report

**Published:** 2016-03

**Authors:** Roopa DA, Shinkhala Singh, Ira Gupta, Saumiya Gopal

**Affiliations:** 1 Dept. of Periodontics, Rama Dental College and Research center, Kanpur, India.; 2 Postgraduate Student, Dept. of Periodontics, Rama Dental College and Research Center, Kanpur, India.; 3 Senior Lecturer, Rama Dental College and Research Center, Kanpur, India.; 4 Reader, Dept. of Periodontics, Rama Dental College and Research Center, Kanpur, India.

**Keywords:** Gingival enlargement, Jones syndrome, Gingivectomy

## Abstract

Gingival enlargement can be caused by a variety of etiological factors like inflammation, drugs, and systemic diseases or can be presented as a part of a syndrome. One such syndrome is Jones Syndrome, which is associated with gingival enlargement and progressive hearing loss. We present here a case of fifteen-year-old boy with gingival enlargement, hearing loss, and generalized alveolar bone loss and diagnosed as Jones syndrome. The diagnosis was made based on history, clinical, radiographic, and histopathological findings. Gingival enlargement was surgically managed using gingivectomy and no recurrence was observed. The patient showed remarkable esthetical and functional improvement.

## Introduction

Gingival enlargement is a broad term that refers to gingival overgrowth without cause suggestion.[1]According to the etiologic factors and pathologic changes, there are varieties of gingival enlargements including inflammatory enlargement, drug-induced gingival enlargement, enlargements associated with systemic diseases, neoplastic enlargement and false enlargement.[2] Gingival enlargement may present in some genetic disorders. According to the etiology, clinical presentation, and histopathological findings, these genetic disorders fall into the four main categories of hereditary gingival fibromatosis (HGF), lysosomal storage disorders, vascular disorders, and disorders associated with dental abnormalities.[1]

HGF, which represents a heterogeneous group of disorders characterized by progressive enlargement of the gingiva may appear as an isolated entity, i.e., as autosomal dominant gingival fibromatosis or as part of a syndrome. One of the many syndromes associated with gingival fibromatosis is Jones syndrome which is characterized by gingival fibromatosis with progressive sensorineural deafness.[1] The literature review did not show many cases of Jones syndrome.[3] The present manuscript presents a case of variant form of Jones syndrome which involved us in a diagnostic dilemma.

## Case Report

 A 15-year-old male patient visited the outpatient Department of Periodontics, Rama Dental College, Kanpur, with the chief complaint of swollen gums involving all his teeth and an inability to chew food. The patient’s gums had progressively enlarged over the last 3-4 months and had pain during mastication and brushing teeth. Medical history of the patient revealed mental retardation and progressive hearing loss. None of his family members had mental retardation, gingival overgrowth, or any other periodontal disease. His parents were not related and there was no history of deafness in his family. A thorough history revealed that the patient started consuming gutka (a form of smokeless tobacco) since 3-4 months ago and was taking up to 2-3 packets a day.

**Figure 1:**
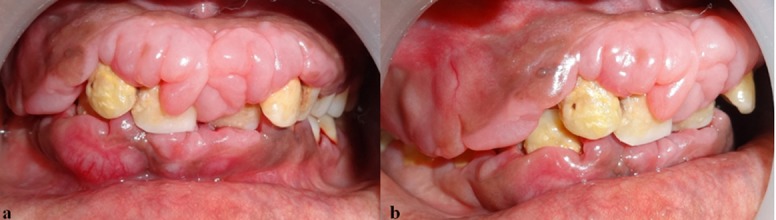
a: Pre-operative frontal view of the gingival enlargement  b: Pre-operative lateral view of the gingival enlargement

Extra-oral examination revealed an apparently symmetrical face and the lips were incompetent. Intra-oral examination showed gingival enlargement in relation to upper and lower anterior teeth involving the interdental papilla, marginal gingiva, and a portion of the attached gingiva. The enlarged gingiva appeared firm, nodular and fibrous. Gingiva was slightly erythematous, presenting grade I bleeding. Plaque and calculus was covering more than half of the crown portion (Figure 1a and 1b).

Severe massive gingival enlargement was covering the entire crown of the right maxillary posteriors, extending from the first premolar to the last molar. The gingival enlargement in this region was reddish-pink in color and was fibro-edematous in consistency. The peculiar feature of enlargement was that it seemed to arise near the mucogingival junction and a flap of enlarged tissue covered the whole of crown portion in the posterior region. Some portion of the flap was attached in the interdental papilla and portions of attached gingiva could be seen. The flap of enlarged gingiva could be raised to see the amount of calculus and plaque deposited on the tooth surface.

**Figure 2:**
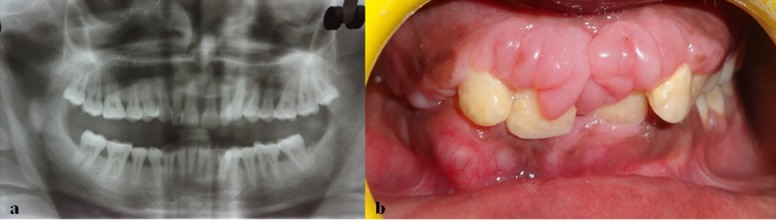
a: Panoramic radiograph showing generalized alveolar bone loss  b: After scaling and root planning

On probing, pseudo-pocket formation was observed. The panoramic radiograph revealed generalized horizontal alveolar bone loss in maxillary and mandibular arches (Figure 2a). The patient’s routine blood examination was found to be normal.

After thorough scaling and root planing the patient was reviewed for one month for any regression of the enlargement. There was no regression of the enlargement but signs of inflammation were reduced (Figure 2b).

**Figure 3:**
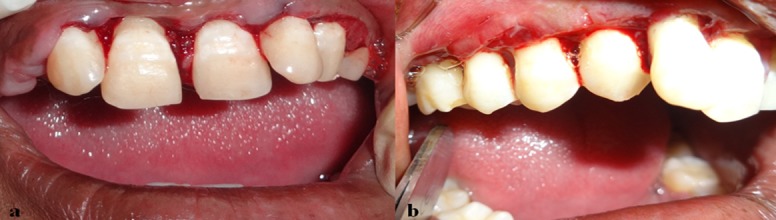
a: Frontal view after gingivectomy  b: Lateral view after gingivectomy

The case was then posted for surgery, and external bevel gingivectomy was performed to excise the gingival enlargement (Figures 3a and 3b). 

The excised tissue was sent for histopathological examination. The postoperative healing was good and the patient was instructed to follow thorough oral hygiene.

**Figure 4:**
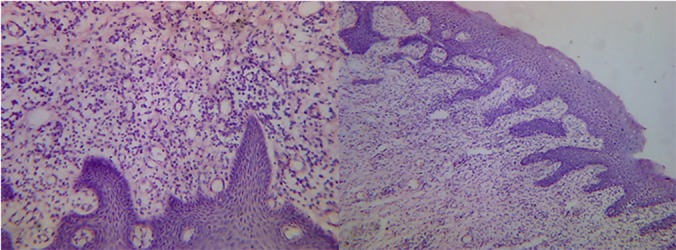
Histopathology of the gingival enlargement

The excised soft tissue from the buccal and interdental areas during surgery was immediately fixed in 10% buffered formaldehyde solution and sent for histopathologic examination. The evaluation of the specimen under light microscope revealed parakeratinized stratified squamous acanthotic epithelium with long thin rete ridges extending into the connective tissue. The underlying connective tissue showed dense wavy bundles of collagen fibers containing numerous fibrocytes and fibroblasts. Some sections in the connective tissue exhibited infiltration of chronic inflammatory cells, a few scattered multinucleated giant cells, and areas of neovascularization that had red blood corpuscles within the lumen of the blood vessels. (Figures 4)

Based on clinical, radiographic, and histopathological findings, the case was diagnosed as idiopathic gingival enlargement with a pronounced inflammatory component. The patient was referred to a general physician to rule out systemic involvement. The case was diagnosed as Jones syndrome by the physician. He was then referred to an ENT surgeon for evaluation of his hearing loss and was diagnosed of having progressive sensory hearing loss. The final diagnosis of the case was gingival fibromatosis associated with Jones syndrome.

**Figure 5:**
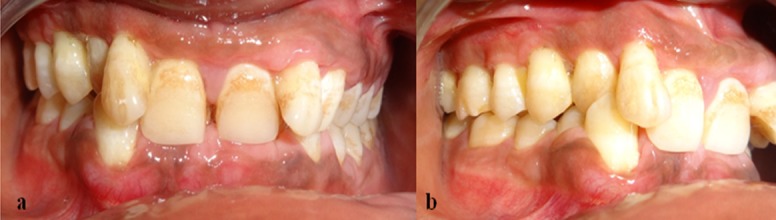
a: Postoperative frontal view  b: Postoperative lateral view

The patient was reviewed after 1, 3, and 6 months to check for recurrence. The postoperative period was uneventful and oral hygiene maintenance was good. No recurrence was observed in this period. The patient was then put into maintenance phase with regular follow-ups. (Figures 5a and 5b)

Gingival enlargement is the overgrowth of the gingiva characterized by an expansion and accumulation of the connective tissue, with or without increased number of cells.[4]It is caused by several factors such as inflammation, leukemia, drugs, and inheritance. The inheritance condition in which the gingival tissue enlarges spontaneously and progressively is identified as HGF.[5] It clinically develops as a slowly progressive, benign, localized or generalized enlargement of keratinized gingiva that, in severe cases, may cover the crowns of the teeth. Enlarged gingiva may be normal in color or erythematous and are firm and nodular on palpation. Although the alveolar bone is usually unaffected, gingival enlargement results in pseudo-pocket formation and periodontal problems due to the difficulties in maintaining an effective level of oral hygiene. The overgrowth may also result in functional and esthetic concerns, create diastema, impede or delay in tooth eruption, and induce changes in facial appearance as a result of lip protrusion.[1] Gingival fibromatosis can occur as an isolated feature or as a part of multisystem disorder. The associated syndromes are Zimmermann-Laband syndrome, Rutherford syndrome.

## Discussion

Cross syndrome, Ramon syndrome, Schinzel-Giedion syndrome, Costello syndrome, Jones syndrome, and so on[1, 6] Jones syndrome (OMIM#135550) is autosomal dominant in inheritance. It is mainly characterized by gingival fibromatosis with progressive sensorineural deafness (Kasaboglu *et al.*, 2004).[7] The described case was in accordance with the features of Jones syndrome.

HGF is traditionally considered an autosomal dominant disease.[8]However, some reports, in which HGF was transmitted as an autosomal recessive inheritance, demonstrated that gingival overgrowth was associated with other systemic conditions, suggesting a syndrome.[9-10] Since the patient in our case report had no family history of gingival overgrowth, mental retardation, or hearing loss suspecting presence of a syndrome in the patient, a diagnosis of Jones syndrome was arrived at as the patient had progressive hearing loss.

There were variations in the gingival enlargement in the current case, presenting severe massive enlargement in the maxillary right posterior region and hardly any enlargement was seen on the left side of the maxillary arch and posterior aspect of mandibular arch. Usually the clinical presentation is generalized involvement of teeth. Another interesting aspect of this case was the presence of generalized horizontal bone loss. This may be attributed to the presence of local factors inducing inflammation and bone loss. There are very few reports of idiopathic gingival enlargement associated with aggressive periodontitis in the literature.[11]

During the diagnosis of the case, the possibility of deleterious effects of gutka was also considered. But the patient was not a massive gutka chewer and was not in the habit of placing the gutka in the lower posterior vestibule, which is the usual area of gutka placement. He did not present any oral lesions associated with gutka consumption, and also no noticeable stain which is usually associated with gutka. Moreover, gutka consumption is not associated with gingival overgrowth per se- and periodontal findings seen in gutka chewers are mainly recession and attachment loss with reduced gingival inflammation.[12]

## Conclusion

Gingival fibromatosis associated with Jones syndrome is a rare entity and our case report presented the diagnosis and management of a patient with Jones syndrome. The case was surgically treated and there was no recurrence in his six- month follow-up visits. 
